# Neuronal Ablation of CoA Synthase Causes Motor Deficits, Iron Dyshomeostasis, and Mitochondrial Dysfunctions in a CoPAN Mouse Model

**DOI:** 10.3390/ijms21249707

**Published:** 2020-12-19

**Authors:** Ivano Di Meo, Chiara Cavestro, Silvia Pedretti, Tingting Fu, Simona Ligorio, Antonello Manocchio, Lucrezia Lavermicocca, Paolo Santambrogio, Maddalena Ripamonti, Sonia Levi, Sophie Ayciriex, Nico Mitro, Valeria Tiranti

**Affiliations:** 1 Unit of Medical Genetics and Neurogenetics, Fondazione IRCCS Istituto Neurologico Carlo Besta, 20126 Milan, Italy; chiara.cavestro@istituto-besta.it (C.C.); antonello.manocchio@istituto-besta.it (A.M.); lucrezia.lavermicocca93@gmail.com (L.L.); 2 DiSFeB, Dipartimento di Scienze Farmacologiche e Biomolecolari, Università degli Studi di Milano, 20133 Milan, Italy; silvia.pedretti@unimi.it (S.P.); simona.ligorio@unimi.it (S.L.); nico.mitro@unimi.it (N.M.); 3 Institut des Sciences Analytiques, Univ Lyon, CNRS, Université Claude Bernard Lyon 1, UMR 5280, 5 rue de la Doua, F-69100 Villeurbanne, France; tingting.fu@univ-lyon1.fr (T.F.); sophie.ayciriex@univ-lyon1.fr (S.A.); 4 Division of Neuroscience, IRCCS San Raffaele Scientific Institute, 20132 Milan, Italy; santambrogio.paolo@hsr.it (P.S.); ripamonti.maddalena@hsr.it (M.R.); levi.sonia@hsr.it (S.L.); 5 Vita-Salute San Raffaele University, 20132 Milan, Italy

**Keywords:** neurodegeneration, CoPAN (COASY protein-associated neurodegeneration), NBIA (neurodegeneration with brain iron accumulation), coenzyme A, iron, mitochondria, mouse model

## Abstract

COASY protein-associated neurodegeneration (CoPAN) is a rare but devastating genetic autosomal recessive disorder of inborn error of CoA metabolism, which shares with pantothenate kinase-associated neurodegeneration (PKAN) similar features, such as dystonia, parkinsonian traits, cognitive impairment, axonal neuropathy, and brain iron accumulation. These two disorders are part of the big group of neurodegenerations with brain iron accumulation (NBIA) for which no effective treatment is available at the moment. To date, the lack of a mammalian model, fully recapitulating the human disorder, has prevented the elucidation of pathogenesis and the development of therapeutic approaches. To gain new insights into the mechanisms linking CoA metabolism, iron dyshomeostasis, and neurodegeneration, we generated and characterized the first CoPAN disease mammalian model. Since CoA is a crucial metabolite, constitutive ablation of the *Coasy* gene is incompatible with life. On the contrary, a conditional neuronal-specific *Coasy* knock-out mouse model consistently developed a severe early onset neurological phenotype characterized by sensorimotor defects and dystonia-like movements, leading to premature death. For the first time, we highlighted defective brain iron homeostasis, elevation of iron, calcium, and magnesium, together with mitochondrial dysfunction. Surprisingly, total brain CoA levels were unchanged, and no signs of neurodegeneration were present.

## 1. Introduction

Two autosomal-recessive inborn errors of coenzyme A (CoA) metabolism, namely PKAN (OMIM: #234200) and CoPAN (OMIM: #615643), are responsible for distinct, albeit clinically overlapping, forms of neurodegeneration with brain iron accumulation (NBIA), a heterogeneous group of highly invalidating neurodegenerative diseases having as a common denominator cerebral iron accumulation. No disease-modifying treatments are available for both PKAN and CoPAN, and management is supportive, aimed at palliation and symptom control. CoA is an essential cofactor found in all living organisms that participates in hundreds of cellular enzymatic reactions, involved in energy and fatty acid metabolism, and regulating numerous biological processes, such as cell growth, cell death, autophagy, epigenetics, signal transduction, protein acetylation, and others [1]. The majority of intracellular CoA is synthetized de novo from pantothenate (vitamin B_5_), ATP, and cysteine, through a highly conserved five-step pathway. Mutations in two of the involved genes, *PANK2* and *COASY*, have been associated to PKAN [2] and CoPAN [3], respectively. *PANK2* encodes one of the four human pantothenate kinase enzymes (PANK1 to PANK4), which is specifically located in the mitochondrial inter-membrane space, catalyzing the first limiting step in CoA biosynthesis. *COASY* encodes for the CoA synthase, a bifunctional enzyme catalyzing the last two, also limiting reaction in the CoA biosynthetic pathway. While PKAN represents a large fraction of NBIA cases, CoPAN appears rarer, being so far identified in few individuals worldwide [4]. These two disorders are characterized by early onset dystonic and parkinsonian features, cognitive impairment, and brain iron accumulation in the basal ganglia [5]. More recently, mutations in *PPCS* encoding for the second enzyme in the CoA biosynthetic pathway have been described in two unrelated families showing a very severe pediatric phenotype characterized by dilated cardiomyopathy and early death without neurodegenerative nor iron overload signs [6]. The presence of defects in *PANK2* and *COASY* in specific subtypes of NBIA obviously suggests a central role of CoA metabolism in neural cell development and maintenance, although the pathogenic mechanisms underlining this connection are not defined yet. Moreover, although iron accumulation characterizes both PKAN and CoPAN, its relationship with CoA dysfunctional biosynthesis or neurodegeneration is still not clear. Furthermore, CoA deficiency has not been formally demonstrated in the central nervous system (CNS) of human PKAN or CoPAN patients.

If there has been a great effort to investigate the molecular, biochemical, and physiophatological basis of PKAN, very little has been done for CoPAN. In humans, *COASY* is located on chromosome 17 and encodes a 564-amino acid enzyme that has two catalytic domains, such as 4′-phosphopantetheine adenylyltransferase (PPAT) and dephospho-CoA (dpCoA) kinase (DPCK), and is strongly activated by phospholipids, such as phosphatidylcholine and phosphatidylethanolamine [7]. The protein has been found mainly in the mitochondrial matrix [3,8] or anchored to outer mitochondrial membranes, exposing both enzymatic domains to the cytosol [7], although a free cytoplasmic and nucleic localization has been reported [9]. Mutations in *COASY* are associated to a severe phenotype characterized by mild oro-mandibular dystonia with dysarthria, spastic-dystonic gait, severe parkinsonism, areflexia in the lower limbs, and loss of the capacity to ambulate independently. Hypointensity in the globus pallidus (GP) or bilateral hyperintensity and swelling of the caudate nucleus, putamen, and thalamus were found on patients’ brain MRI, along with increased free carnitine and decreased acylcarnitines in the patients’ blood samples [3,10,11]. Analysis of fibroblasts derived from CoPAN patients showed impaired, but not completely abolished, de novo synthesis of CoA and dpCoA. This would suggest the presence of alternative routes for CoA production, recycling, or exchange, as well as the preservation of a residual catalytic activity of the mutant CoA synthase protein [3]. More recently, mutations in *COASY* associated with the complete absence of the protein were reported in two cases of pontocerebellar hypoplasia, microcephaly, and arthrogryposis with an invariable perinatal lethal phenotype [12]. Given the rarity of the disease, only very few CoPAN models have been generated so far. In yeast, CoA synthesis is carried out by five sequential enzymes (CAB1 to CAB5), and PPAT and DPCK activities reside on different proteins encoded by *CAB4* and *CAB5*, respectively. Deletion of both genes results in a lethal phenotype, which is rescued by re-expression of human COASY protein. Moreover, mutant COASY expression leads to a phenotype characterized by auxotrophy for panthotenate, reduced growth, decreased amount of CoA in isolated mitochondria, impairment of mitochondrial respiration, and dysfunction of iron homeostasis and lipid content [3,13]. In Zebrafish, the morpholino-mediated complete ablation of *coasy* expression leads to severe alteration of development and premature death, while the partial downregulation resulted in a milder phenotype, characterized by a generalized reduction in size and poor definition of CNS structures and vasculature arborization, as well as a CoA level reduction in embryos. Furthermore, the expression of bone morphogenetic protein (Bmp) receptors and their activity were decreased, while cell death increased [14].

To date, no CoPAN mammalian animal model has been generated. The available *Pank2*-null mouse showed male infertility due to azoospermia [15]; impaired mitochondrial function in older animals [16]; regional perturbations in CoA metabolism, iron homeostasis, and dopamine metabolism; and functional defects of complex I and pyruvate dehydrogenase in GP [17]. However, it did not suffer from movement disorders and had no signs of neurodegeneration, implying that, at least in mice, the other *Pank* genes may partially compensate for *Pank2* loss. Since *COASY* is the only gene so far known to encode for CoA synthase in mammals, we hypothesized that a mouse model defective for this enzyme could display a pathological phenotype. To gain new insights into the pathogenic mechanisms of neurodegeneration and iron homeostasis deregulation in CoPAN, we generated a conditional neuronal-specific *Coasy* mouse knock-out (KO) model (Syn-Coasy), as driven by the *synapsin1-Cre* transgene. This model consistently developed a severe early onset neurological phenotype characterized by sensorimotor defects and dystonia-like movements, early death, impairment of iron homeostasis, and mitochondrial dysfunction. Remarkably, we did not detect significantly variations in brain CoA levels nor signs of neuropathology.

## 2. Results

### 2.1. Generation of the Syn-Coasy Mouse Model

**Figure 1:**
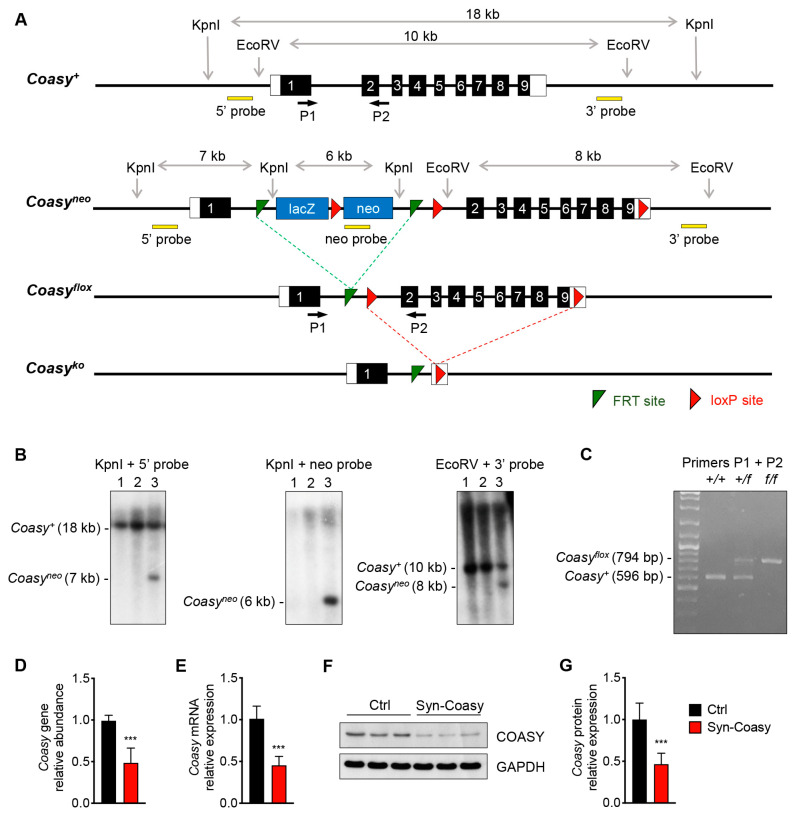
Generation of Cre-loxP Coasy transgenic mice. (**A**) A schematic diagram of the Coasy wild-type locus (*Coasy^+^*) Homologous recombination of the targeting construct resulted in production of the *Coasy^neo^* allele. By sequential mating with FLPE and Cre transgenic mice, *Coasy^flox^* and *Coasy^ko^* alleles were obtained. The primers (black arrows) used for PCR genotyping, as well as the probes (yellow boxes) used for southern blot analysis are shown. (**B**) Southern blot analysis on KpnI/EcoRV-digested genomic DNA of ES cell clones with 5′, neo and 3′ probes, showing wild-type (*Coasy^+^*) and recombinant (*Coasy^neo^*) bands. Lane 1 and 2: *Coasy^+/+^*; lane 3: *Coasy^+/neo^*. (**C**) PCR genotyping strategy using primers P1 and P2 to distinguish the wild-type (*Coasy^+^*) and the floxed (*Coasy^flox^*) alleles on wild-type (+/+), heterozygous (+/f), and homozygous (f/f) recombinant mice. (**D**) Relative amount of Coasy gene in the brain of Ctrl (*n* = 8) and Syn-Coasy (*n* = 8), tested by RT-qPCR. (**E**) Relative Coasy mRNA expression in Ctrl (*n* = 8) and Syn-Coasy (*n* = 8) mice brain. (**F**) Western blot analysis and (**G**) densitometric quantification of Coasy in the brain from Ctrl (*n* = 8) and Syn-Coasy (*n* = 8) mice. GAPDH was used as the loading control. For (**D**,**E**,**G**), mean ± SD is shown. *** *p* < 0.001 (Student’s *t*-test).

Using homologous recombination, we generated floxed mice in which the region encompassing exons two to nine of the *Coasy* gene is flanked by loxP sites (Coasy^flox^) (Figure 1A). First, homozygous floxed animals were crossed with a transgenic mouse expressing the Cre recombinase in the germ line. While heterozygous mice were viable and normal, we found that constitutive ablation of Coasy is not compatible with life. Indeed, out of 62 pups born from a cross between heterozygous mice, none were found to be homozygous for Coasy deletion (expected: 15,5). Since CoPAN is characterized by neurodegeneration in humans, we generated a conditional neuronal-specific Coasy-deleted model by crossing Coasy^flox/flox^ with a transgenic mouse expressing the Cre under the control of the rat synapsin 1 (*Syn1*) promoter. The *Syn1-Cre* transgene becomes active in the developing CNS at embryonic day 12.5 (E12.5) [18], representing a commonly used strategy for neuron-specific loss-of-function experiments. To confirm transgene activity, we also crossed Syn1-Cre transgenic mouse with R26-stop-YFP reporter mice carrying a fluorescent YFP protein downstream of an loxP-flanked STOP cassette. We confirmed that the transgene was strongly activated through all the brain (Figure S1).

To analyze the *Coasy* gene deletion in Coasy^flox/flox^;+/Syn1-Cre mouse (hereafter Syn-Coasy), we performed real-time quantitative PCR (RT-qPCR) analysis on DNA extracted from total brain that confirmed a 50% inactivation of the gene (Figure 1D). Accordingly, we also measured a reduction of about 50% of Coasy transcript expression in the brain of Syn-Coasy mice by RT-qPCR (Figure 1E). Western blot analyses also demonstrated a 50% reduction of COASY protein, as compared to the control (Ctrl) littermates (Figure 1F,G), while no differences in protein levels were observed in extra-CNS tissues (Figure S2).

### 2.2. Clinical and Pathological Characterization of the Syn-Coasy Mouse Model

**Figure 2:**
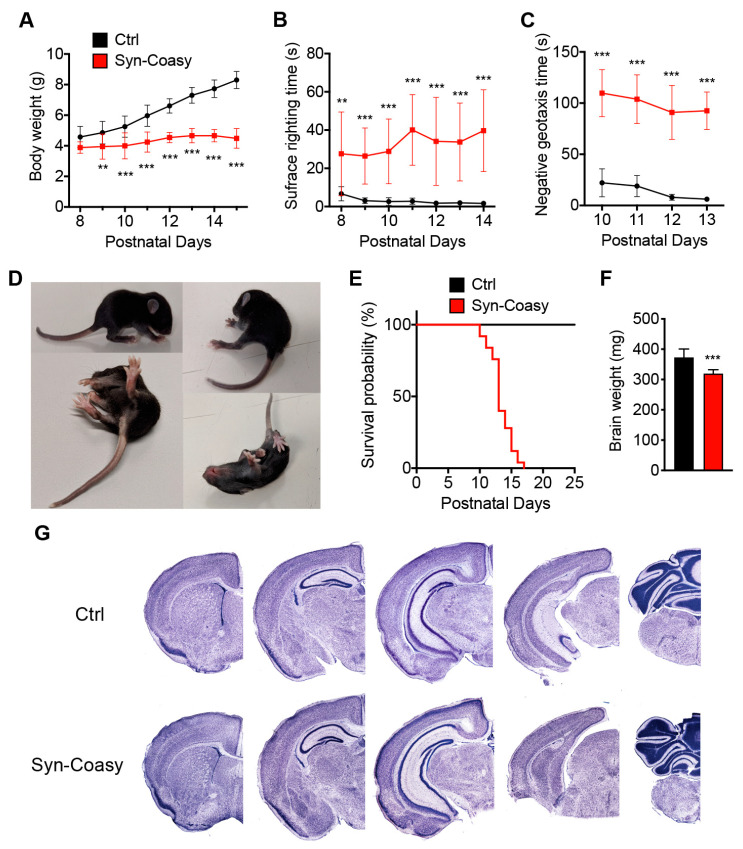
Clinical and pathological characterization of Syn-Coasy mice. (**A**) Body weights variation, (**B**) surface righting, and (**C**) negative geotaxis analysis in Ctrl (*n* = 14) and Syn-Coasy (*n* = 14) mice. ** *p* < 0.01; *** *p* < 0.001 (one-way ANOVA). (**D**) Images of a 13-day-old Syn-Coasy mouse showing postural defects and dystonic behaviors. (**E**) Kaplan–Meier survival probability in Syn-Coasy (*n* = 20) mice. (**F**) Brain weight of Ctrl (*n* = 8) and Syn-Coasy (*n* = 8) mice. *** *p* < 0.001 (Student’s *t*-test). (**G**) Coronal sections stained with tionin showing no obvious alteration trough the forebrain of mutant (bottom panels) mice compared to normal (top panels) littermates. For all charts, mean ± SD is shown.

Pups from heterozygous parents showed a gender and genotype distribution compatible with a mendelian autosomal recessive trait with no evidence of reduced viability. Although Syn-Coasy mice were indistinguishable from their Ctrl littermates during the first few days of life, they showed growth arrest starting approximately from postnatal day 8 (P8) (Figure 2A) in conjunction with the appearance of a sensorimotor phenotype assessed by surface righting (Figure 2B) and negative geotaxis (Figure 2C) tests.

Within a couple of days, Syn-Coasy mice showed a very severe phenotype, characterized by movement alterations with abnormal and generalized dystonia-like movements, loss of postural equilibrium, flexing or prolonged stiff extension of the limbs and rigidity of the tail, involuntary twisting movements, and abnormal tail suspension reflexes (Figure 2D, Video S1). To avoid unnecessary suffering of animals, early compassionate euthanasia was performed at around P13 (Figure 2E).

Syn-Coasy mice were observed suckling through all life and, at the time of euthanasia, milk was present in their stomachs. No altered phenotype was observed in Coasy^+/flox^;+/Syn1-Cre or Coasy^flox/flox^ littermates; therefore, all of them were considered as controls in this study. Moreover, male and female Syn-Coasy animals showed a similar lifespan and timing of phenotype manifestation. Although brains collected from Syn-Coasy mice were significantly smaller than those from Ctrl littermates (Figure 2F), they did not show any gross alteration (Figure 2G), nor astrocytosis or neuroinflammation (Figure S3). Moreover, we did not observe neuronal loss or the presence of intracellular inclusions on histological and immunohistochemical analysis (not shown).

### 2.3. CoA Biosynthesis and Protein Acetylation

**Figure 3:**
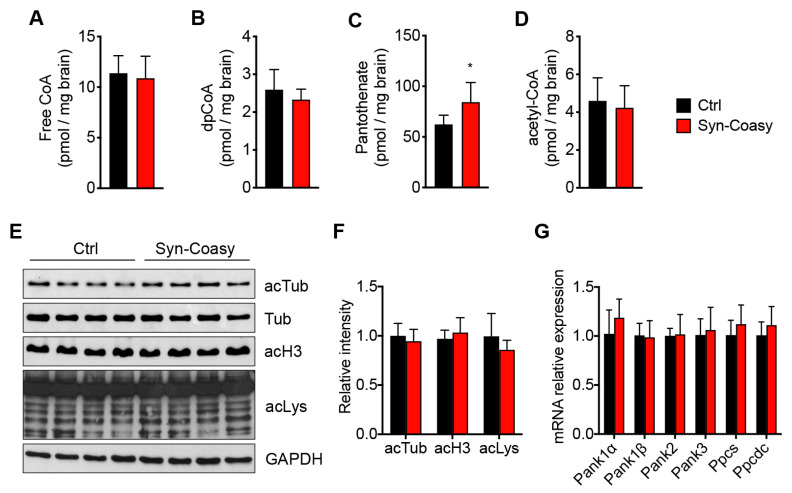
CoA biosynthesis and protein acetylation. Mass spectrometry quantification of (**A**) free CoA, (**B**) dpCoA, (**C**) pantothenate, and (**D**) acetyl-CoA in the forebrain from Ctrl (*n* = 6) and Syn-Coasy (*n* = 6) mice (age P12–P14). * *p* < 0.05 (Student’s *t*-test). (**E**) Western blot analysis and (**F**) densitometric quantification of acetylation levels for tubulin (acTub), histone 3 (acH3), and total lysine residues (acLys) in forebrain homogenates from Ctrl (*n* = 8) and Syn-Coasy (*n* = 8) mice (age P12–P14). GAPDH was used as the loading control for acH3 and acLys, while acTub was normalized against total tubulin (Tub). No statistically significant differences were observed (one-way ANOVA). (**G**) Relative expression of Pank1α and β, Pank2, Pank3, Ppcs, and Ppcdc mRNAs in the Ctrl (*n* = 5) and Syn-Coasy (*n* = 5) mice forebrain. No statistically significant differences were observed (one-way ANOVA). For all charts, mean ± SD is shown.

Since COASY is the only known bifunctional enzyme catalyzing the final two steps of CoA biosynthesis, we measured by mass spectrometry the levels of CoA, dpCoA, and pantothenate in the cerebrum of P12–P14 Syn-Coasy and Ctrl mice. Surprisingly, we did not observe any statistically significant difference in the amount of CoA and dpCoA (Figure 3A,B), while a significant increase of pantothenate was observed (Figure 3C). Given that acetyl-CoA is one of the most abundant CoA derivatives and the main intracellular acyl donor, and CoA/acetyl-CoA amounts determine protein acetylation levels, we quantified acetyl-CoA, as well as protein acetylation, in the Syn-Coasy and control brains. Consistent with the unchanged level of CoA, we observed an unaltered acetyl-CoA (Figure 3D) amount and protein acetylation levels of total lysine residues (acLys), α-tubulin (acTub), and histone 3 (acH3) (Figure 3E,F). In order to exclude a compensatory mechanism, we analyzed the mRNA expression of genes in the CoA synthetic pathway upstream of Coasy, such as Pank1α and β, Pank2, Pank3, Ppcs, and Ppcdc. None of the above genes showed a different expression in the cerebrum of Syn-Coasy as compared to Ctrl mice (Figure 3G).

### 2.4. Iron Content and Homeostasis

**Figure 4:**
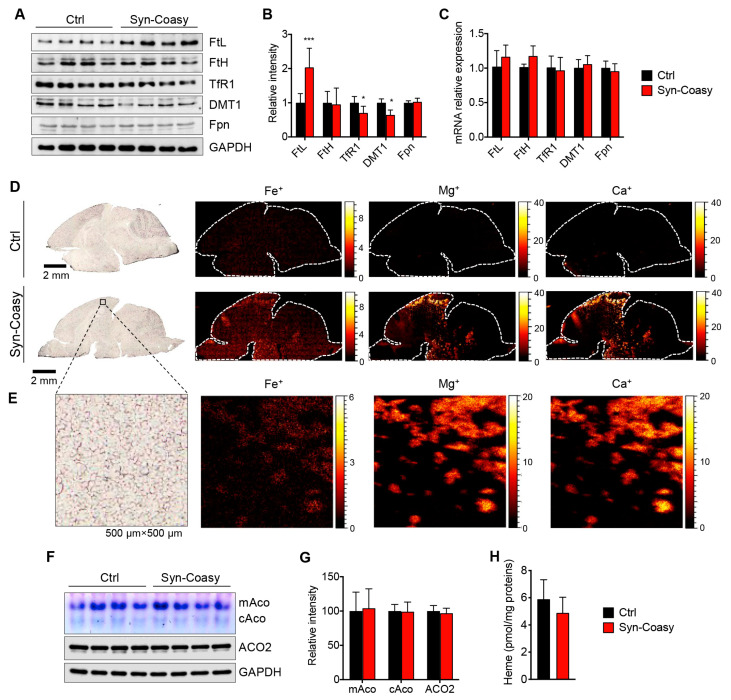
Iron content and homeostasis analysis. (**A**) Immunoblot and (**B**) densitometric quantification of proteins involved in homeostasis in forebrain homogenates from Ctrl (*n* ≥ 8) and Syn-Coasy (*n* ≥ 8) mice (age P12–P14). GAPDH was used as the loading control. * *p* < 0.05; *** *p* < 0.001 (one-way ANOVA). (**C**) Relative expression of the genes encoding the proteins analyzed in (**A**), in the Ctrl (*n* = 5) and Syn-Coasy (*n* = 5) mice forebrain. No statistically significant differences were observed (one-way ANOVA). (**D**) Elevation of Fe, Ca, and Mg in Syn-Coasy compared to Ctrl by TOF-SIMS imaging. (**E**) The high-resolution imaging of the cortex (500 µm × 500 µm) of the Syn-Coasy brain clearly showed colocalization of the Fe^+^, Ca^+^, and Mg^+^. (**F**) Representative in-gel activity of mitochondrial (mAco) and cytosolic (cAco) aconitase, and immunoblot of mitochondrial aconitase (ACO2) in forebrain homogenates from Ctrl (*n* = 8) and Syn-Coasy (*n* = 8) mice. (**G**) Densitometric quantification of (**F**). GAPDH was used as the loading control. (**H**) Heme quantification by absorbance at 400 nm of the soluble forebrain lysates. For all charts, mean ± SD is shown.

The main pathologic hallmark of CoPAN is iron overload in the brain, which probably precedes the onset of neurological symptoms. Although we did not observe evident iron accumulation in the brain of Syn-Coasy animals by classical histological Perl’s staining (not shown), we found a consistent statistically significant alteration in the steady-state levels of proteins involved in controlling cellular iron homeostasis. Specifically, we observed a considerable increase of the light chain of cytoplasmic ferritin (FtL), which in combination with the heavy chain (FtH) forms the main complex that stores iron in the cell (Figure 4A,B). Moreover, we found a reduction of both transferrin receptor 1 (TfR1), the membrane glycoprotein that imports iron into the cell by internalizing the transferrin-iron complex through receptor-mediated endocytosis, and of divalent metal transporter 1 (DMT1), which pumps iron out of the endosome into the cytosol (Figure 4A,B).

No changes in the levels of the ferritin heavy chain, also quantitatively confirmed by specific ELISA (Figure S4), as well as of the only know cellular iron exporter ferroportin (Fpn) were observed (Figure 4A,B). Levels of FtL, FtH, Fpn, DMT1, and TfR1 proteins are finely post-transcriptionally regulated by iron-regulatory proteins (IRPs), IRP1 and IRP2. At a low intracellular iron concentration, IRPs bind the 3′ UTR of TfR1 and DMT1 mRNAs, preventing them from degradation and allowing cells to import more iron; meanwhile, they also bind the 5′ UTR of ferritins and ferroportin mRNAs, inhibiting their synthesis. Contrariwise, in high-iron conditions, IRP1 is converted into aconitase and IRP2 is degraded, promoting iron storage and limiting iron entry [19]. The evidence that FtL, FtH, and Fpn transcripts were unchanged between Syn-Coasy and Ctrl brains (Figure 4C) agrees with the above mechanisms, suggesting that we are probably observing precocious signs of intracellular iron accumulation in the brain of recombinant animals. To corroborate this hypothesis, we performed time of flight-secondary ion mass spectrometry (TOF-SIMS) imaging analysis on heat-treated sagittal brain sections. A slight, albeit evident, diffuse iron accumulation was found in Syn-Coasy mice, compared to control littermates (Figure 4D,E). Interestingly, we also observed conspicuous accumulation of calcium and magnesium that spatially overlaps the iron-enriched regions, as illustrated by the whole section mapping as well as by high-resolution imaging of selected cortex regions (Figure 4D,E; Figure S5).

In the cell, iron is mainly used in two iron-dependent biosynthetic pathways to form iron-sulfur clusters (Fe-S) and heme, and succinyl-CoA is an essential precursor for heme biosynthesis. In order to verify if neuronal Coasy deficiency leads to impairment of these pathways, we analyzed the activity of two Fe-S-containing enzymes and heme content in Syn-Coasy brain homogenates. In-gel activities of cytosolic and mitochondrial aconitase (cAco and mAco) showed no differences between control and Syn-Coasy animals (Figure 4F,G). Likewise, spectroscopic quantification revealed a reduction of heme in the Syn-Coasy brain compared to control littermates despite not being statistically significant (Figure 4H).

**Figure 5:**
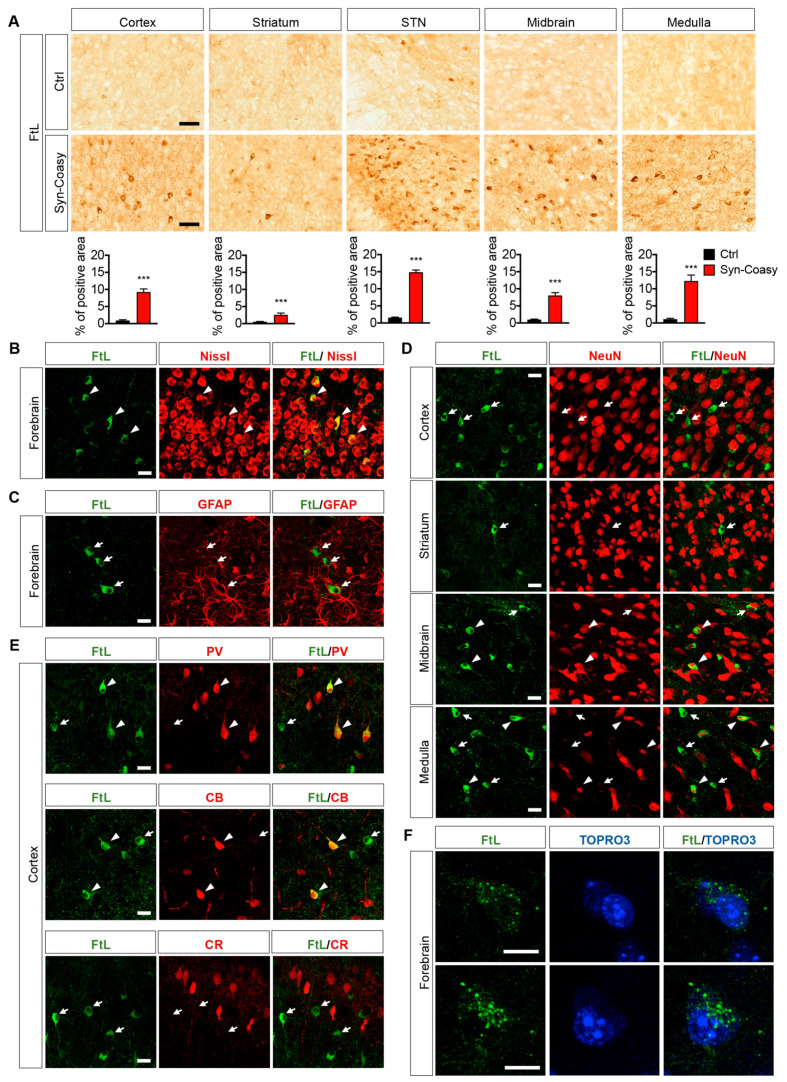
L-chain ferritin accumulation in sections of the Syn-Coasy mouse brain. (**A**) Immunohistochemical analysis of brains of 12–14-day-old animals showing FtL-positive cells accumulation in the cortex, striatum, subthalamic nucleus (STN), midbrain, and medulla of Syn-Coasy mice (bottom panels; *n* = 6) compared to control littermates (top panels; *n* = 6). Bar charts showing quantification of the percentage of the area occupied by FtL-positive staining are reported as mean ± SD. *** *p* < 0.001 (Student’s t-test). (**B**) Representative confocal immunofluorescence on the Syn-Coasy mice forebrain performed with an FtL antibody (green) and neuronal Nissl staining (neurotrace, red). Arrowheads indicate co-localization. (**C**) Double staining of FtL (green) and GFAP (red) in the Syn-Coasy forebrain. Arrows indicate an absence of co-localization. (**D**) Co-localization (arrowheads) or absence of co-localization (arrows) of FtL (green) and NeuN (red) in the Syn-Coasy cortex, striatum, midbrain, and medulla. (**E**) Presence (arrowheads) or absence (arrows) of co-localization of FtL (green) and PV, CB, or CR (red) in Syn-Coasy cortical tissue. (**F**) Detail of a Syn-Coasy forebrain co-labeled with FtL (green) and nuclear TOPRO3 (blue) showing granular ferritin accumulation. Scale bars are 100 μm (**A**), 20 μm (**B**–**E**), and 10 μm (**F**).

Immunohistochemical analysis performed on brain coronal sections decorated with an FtL-specific antibody showed a pronounced increase of cytoplasmic ferritin, especially in the sensory and motor cortex, subthalamic nuclei (STN), midbrain, medulla, and, to a lesser extent, in the striatum (Figure 5A). We did not observe any ferritin accumulation in the cerebellum (not shown), although Cre transgene was also expressed in this area (Figure S1). Co-labeling with FtL antibody and Nissl stain on the Syn-Coasy forebrain revealed that ferritin accumulation seems to be mostly confined to neurons (Figure 5B) while GFAP^+^ glial cells did not accumulate ferritin (Figure 5C). Interestingly, while in the cortex and striatum, ferritin-accumulating neurons were negative or only weakly positive for the neuronal marker NeuN, in the midbrain and medulla, most of the ferritin-accumulating cells were NeuN positive (Figure 5D). It has been reported that, in some brain areas, neurons can show very faint or no staining for NeuN. For instance, in the cerebellum, as well as in the dorsal lateral geniculate nucleus (dLGN), NeuN-negative cells coincide with inhibitory GABAergic interneurons [20,21]. Indeed, co-immunolabeling Syn-Coasy forebrain sections with an anti-FtL together with a set of GABAergic-specific markers revealed that the majority of ferritin-accumulating cells in the cortex were parvalbumine (PV)+ and/or calbindin (CB)+ GABAergic interneurons, while calretinin (CR)+ cells do not accumulate ferritin (Figure 5E). Moreover, we found that FtL often accumulates in cytoplasmic granules (Figure 5F). Stored iron is released by the lysosome-autophagy-dependent ferritin degradation through a mechanism termed ferritinophagy by which iron can be released and subsequently reutilized by the cell [22]. To test if ferritin accumulation was due to impairment of autophagy [23], we evaluated steady-state levels of proteins linked to autophagy or lysosomes, such as LC3, p62, Beclin1, or lysosomal-associated membrane protein 1 (LAMP1), in control and Syn-Coasy brains, but we were unable to highlight any significant change (Figure S6).

### 2.5. Mitochondrial Bioenergetics and Morphology

**Figure 6:**
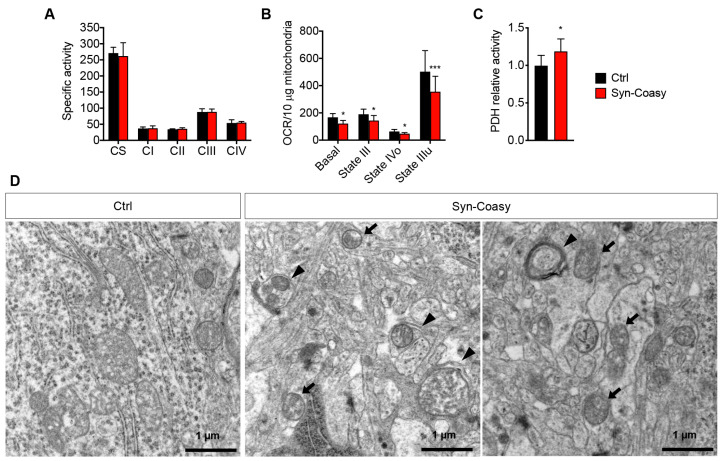
Mitochondrial functions. (**A**) MRC activities in forebrain from Ctrl (*n* = 8) and Syn-Coasy (*n* = 8), expressed as nmoles/min/mg of proteins. CS, citrate synthase; CI-IV, MRC complexes I-IV. No statistically significant differences were observed (one-way ANOVA). (**B**) Pyruvate/malate-dependent oxygen consumption rate (OCR) measured in isolated mitochondria from the Ctrl (*n* = 3) and Syn-Coasy (*n* = 3) forebrain under basal conditions, and after sequential addition of ADP (State III), oligomycin (State IVo), and FCCP (State IIIu). * *p* < 0.05; *** *p* < 0.001 (one-way ANOVA). (**C**) PDH relative activity in the forebrain from Ctrl (*n* = 10) and Syn-Coasy (*n* = 10). * *p* < 0.05 (one-way ANOVA). For all charts, mean ± SD is shown. (**D**) Electron microscopy of the forebrain showing altered mitochondria (arrows) and autophagosomes (arrowheads) in Syn-Coasy mice.

Since COASY protein has been found associated with mitochondria, and these organelles are the major site for CoA storage and utilization, we investigated if neuronal Coasy loss could alter mitochondrial functions. To this aim, we performed spectrophotometric assays to measure the biochemical activity of single mitochondrial respiratory chain (MRC) complexes in forebrain homogenates derived from Syn-Coasy and control mice. We did not observe alteration of complex I, II, III, and IV, and citrate synthase (CS) enzymatic activity (Figure 6A). 

We then investigated whether global mitochondrial respiration was altered in the Syn-Coasy mouse brain by microscale oxygraphy. Mitochondria isolated from the forebrain were incubated with a pyruvate/malate mix as the substrate of TCA cycle/complex I-driven respiration, and the oxygen consumption rate (OCR) was measured under basal conditions, and after sequential addition of ADP, oligomycin, FCCP, and rotenone. This allows assessment of the different states of mitochondrial respiration, such as basal respiration (state II), respiration stimulated by ATP synthesis from ADP and phosphate (state III), respiration due to proton leak in the presence of oligomycin (state IVo), and FCCP uncoupled-stimulated respiration (state IIIu). We found that all the above respiration states were consistently and significantly lower in Syn-Coasy mitochondria as compared with control mice (Figure 6B), indicating an impairment of pyruvate-dependent mitochondrial respiration in the Syn-Coasy mouse brain.

Pyruvate derived from glycolysis is transported into mitochondria and then converted into acetyl-CoA by the pyruvate dehydrogenase complex (PDH) in a reaction that depends on CoA amounts into the mitochondrial matrix. Recently, it has been reported that *Pank2* −/− mice are characterized by decreased PDH activity in the globus pallidus [17], and that impaired CoA homeostasis in *Drosophila* leads to decreased 4′-phosphopantetheinylation of mitochondrial acyl carrier protein (mtACP), resulting in a decrease in lipoic acid synthesis with reduced activity of lipoylated proteins, such as PDH [24]. Conversely, we found normal levels of lipoic acid (Figure S7), and a modest but significant increase of PDH activity in cerebrum homogenates from Syn-Coasy mice compared to controls, suggesting a sort of compensatory effect on bioenergetic metabolism through the increase of TCA cycle feeding (Figure 6C).

Finally, electron microscopy analysis on forebrain semi-thin sections revealed the presence of altered mitochondria with deranged cristae structure, together with various auto- and mitophagosomes and a general membranes disorganization of the brain tissue (Figure 6D).

### 2.6. Evaluation of Oxidative Stress

One of the consequences of impaired mitochondrial respiration is the increase of radical oxygen species (ROS) and thus of oxidative stress. We measured the levels of carbonyl groups, a marker of protein oxidation, by an OxyBlot experiment in the cerebrum of four Syn-Coasy and four control littermates. We did not observe any evident difference in the levels of carbonyls between the two groups (Figure S8). We also performed Western blot and immunohistochemical analysis on the same tissues with an antibody specific for 4-hydroxynonenal (4-HNE), a biomarker for lipid peroxidation also involved in ferroptosis. Again, no significant differences were observed (Figure S8). RT-qPCR showed a slight, albeit statistically significant, increase of the cytosolic superoxide dismutase 1 (Sod1) in the Syn-Coasy cerebrum compared to controls, while no differences in the expression of the mitochondrial Sod2 and of the peroxisomal catalase (Cat) (Figure S8) were observed.

## 3. Discussion

One of the main hallmarks of neurodegeneration associated to inborn errors of CoA metabolism, such as PKAN and CoPAN, is iron accumulation in the brain, but the reason for this phenomenon, as well as the links between CoA metabolism, iron homeostasis, and the neurodegenerative process, are still unknown. These gaps are mainly due to both the rarity of the disorders and the lack of suitable model organisms that faithfully recapitulate the characteristics of human disease. Moreover, most of the models generated so far pertain PKAN, accounting for a large fraction of NBIA cases. Herein, we generated a neuronal-specific null mouse model for *Coasy*, the only known gene encoding for the bifunctional enzyme CoA synthase, catalyzing the last two steps of CoA biosynthesis. We collected evidence that *Coasy* is a gene essential for life since its constitutive ablation is associated with embryo lethality. Contrarywise, neuronal conditional Syn-Coasy KO mice developed a severe early onset phenotype, characterized by dystonia-like movements, abnormal gait, and sensorimotor defects, with a median lifespan of 13 days, but without signs of neuronal loss or developmental defects. With the exception of reduced lifespan, the mouse phenotype recapitulated the human clinical features, although in CoPAN cases, only missense mutations preserving a residual catalytic activity of the mutant CoA synthase protein have so far been described. We still do not know why we did not observe neuronal loss despite the presence of a neurological phenotype and why we did not detect iron accumulation in the globus pallidus, but we could ascribe these phenomenon to the fact that we have generated a mouse model with neuronal-specific Coasy ablation, which does not take into account the contribution of astrocytes to the pathogenesis of the disease. We would probably gain more knowledge on this disorder by generating a mouse model either carrying astrocyte-specific Coasy ablation or a knock-in carrying one of the point mutations described in human patients associated with the maintenance of residual COASY activity. More recently, loss-of-function variants of COASY associated with complete loss of the enzyme were reported in two cases of severe pontocerebellar hypoplasia, prenatal onset microcephaly, and arthrogryposis, with an invariable lethal phenotype in the perinatal period [12]. Moreover, the complete downregulation of *coasy* expression in zebrafish leads to a severe alteration of development, with death occurring within 72 h post fertilization [14].

Strangely enough, despite no other pathway being currently known for de novo CoA synthesis, and COASY being considered a crucial enzyme in both mice and humans, we found normal CoA, dpCoA, acetyl-CoA, and protein acetylation levels in the brain of Syn-Coasy animals. Contrariwise, a significant increase of pantothenic acid was detected, suggesting that the enzymatic block due to Coasy ablation could cause an upstream accumulation of CoA precursors up to pantothenate. It is also possible that the intermediate 4′-phosphopantetheine, which cannot be converted into dpCoA, is diverted into pantothenate, thus triggering a vicious circle leading to pantothenate accumulation. Why did we not observe CoA deficiency in this model? The rationale behind this phenomenon is not known at the moment, but we can hypothesize a couple of possible explanations. First, we specifically ablated *Coasy* in neurons, while the gene was preserved in the other cells, such as astrocytes, oligodendrocytes, and microglia, representing at least half of the cells composing the brain. It is possible that non-neuronal cells try to compensate for neuronal CoA deficiency by increasing their CoA biosynthesis, thus masking CoA deficiency in assays on total brain homogenates. It is well known that the energy requirements of the brain are very high, and tight regulatory mechanisms operate to ensure adequate spatial and temporal supply of energy substrates, which can be transferred from astrocytes to neurons. This is the central point of the astrocyte-neuron lactate shuttle model proposed over a decade ago, according to which the astrocytic lactate overproduced from glycolysis is transferred to neurons and reconverted to pyruvate, in order to sustain neuronal energy production [25]. Despite the fact that the existence of a CoA plasmatic membrane transporter is still debated and the paradigm that charged metabolites, including CoA, are membrane impermeable, the ability of astrocytes and neurons to exchange vesicles containing different factors, metabolites, or even organelles, including mitochondria, has been experimentally demonstrated [26,27]. So, in principle, we cannot exclude that vesicles containing mitochondria with fully functional COASY derived from astrocytes or CoA itself could reach the KO neurons lacking the protein. This is of course a speculative hypothesis, which needs to be experimentally demonstrated.

Second, it has been recently shown that under physiological conditions, brain CoA increased in mice at around P12. A mouse model characterized by the constitutive ablation of *Pank1* together with the neuronal loss of *Pank2* genes displayed normal levels of CoA until P12 but showed reduced CoA in the brain and spinal cord at P19–P21, the time when mice consistently developed neurological symptoms and died [28]. It is possible that the average lifespan of our Syn-Coasy animals prevents us from measuring an effective reduction of CoA in the right temporal window, although it remains unexplained how neurons are able to develop and stay alive until term without the unique cellular CoA synthase enzyme. Finally, the existence of alternative enzymes or biochemical pathways devoted to CoA biosynthesis or the exchange of CoA between different organelles inside the cell could be hypothesized even if, at the moment, not analytically proven.

Recently, Jeong et al. [17] reported a regional *Coasy* downregulation in the globus pallidus of *Pank2*-null mouse model. The authors ascribed this finding to defective CoA metabolism, although they did not directly measure the CoA amount. In parallel, they proposed that CoA was consumed for the phosphopantetheinyl activation of certain proteins. One of these phosphopantetheinylated proteins is mtACP, whose activation failure has been connected to impairment of Fe-S cluster biogenesis, reduction of lipoic acid synthesis, and protein lipoylation [24]. We verified if the same mechanism would work for our CoPAN model, but we failed to measure any alteration of the Fe-S-containing enzymes, such as the mitochondrial respiratory complex I and cytosolic or mitochondrial aconitase, as well as any reduction of lipoic acids or of the activity of the lipoylated PDH enzyme.

Instead, the Syn-Coasy mice accumulate iron in the brain, although at very low levels and in a diffuse manner that does not correlate with the human condition. Interestingly, iron accumulation precisely overlaps with augmented calcium and magnesium levels. Although our knowledge on the molecular mechanisms linking iron with calcium and magnesium accumulation in this disorder is limited, we have previously demonstrated a harmful iron–calcium connection in PKAN iPSCs-derived neurons, as well as brain calcification in both PKAN and CoPAN patients [3,29]. Associated to iron accumulation, here we found a profound and consistent alteration of iron homeostasis-regulating proteins, such as FtL, TfR1, and DMT1. Particularly evident is the accumulation of the ferritin L-chain in different brain areas, which seems to mainly accumulate into GABAergic interneurons of internal cortical layers. Our hypothesis is that the very short lifespan of the animals prevented iron from accumulating to high levels. Nevertheless, FtL accumulation and TfR1 and DMT1 reduction would represent the primordial molecular events of a subsequent more prominent iron dyshomeostasis. This hypothesis is corroborated by the evidence that a normal amount of heme and aconitase activity is present in Syn-Coasy mice euthanized at 13 days of life.

Transferrin-bound iron enters the cell by TfR1 after internalization via clathrin-mediated endocytosis. Next, while iron is released by vesicle acidification, the endosomal system allows the transferrin-TfR1 complex to be recycled to the plasma membrane. TfR1 internalization is reported to be post-translationally regulated by palmitoylation, whereas palmitoylation impairment causes increased TfR1 internalization and iron overload. Alteration of endosome trafficking has been recently proposed as a common mechanism inducing iron dyshomeostasis in different NBIA forms [30]. Further studies are needed to investigate if TfR1 palmitoylation and recycling is altered in our model.

Intriguingly, the reasons why only FtL, and not FtH, accumulates in Syn-Coasy neurons remain unclear. In animals, ferritin is composed of 24 FtL and FtH subunits in ratios that vary in different cell types. FtL and FtH maintain distinct functions: H chains exhibit ferroxidase activity, converting Fe^+2^ to Fe^+3^ so that iron can be stored in the ferritin mineral core, preventing reactions of Fe^+2^ with oxygen and the formation of damaging reactive oxygen species (ROS), while the physically more stable L chains accelerate the transfer of iron from the ferroxidase center to the iron core and improve the overall iron-sequestering process [31]. The ferritins rich in H chains are found predominantly in the heart and brain, and possess a more pronounced antioxidant activity, while the ferritins enriched in L chains of the spleen and liver are able to contain more iron atoms, displaying a more pronounced iron storage function also due to their increased stability [32]. Moreover, it has been observed in vitro that in aging lens epithelial cells, steady degradation of H-chain ferritin contributes to the maintenance of a constant level of this subunit, while the slower turnover of the L chain can result in accumulation of FtL-enriched ferritin associated with cytoplasmic inclusion bodies [33], similar to those observed in neurons differentiated from neuroferritinopathy patient-derived iPSCs [34].

We also provide evidence that loss of neuronal *Coasy* leads to alteration of mitochondrial morphology and energetic functions, probably causing the loss of fully functional neurons observed in human patients. These findings correlate with previous observation showing mitochondrial dysfunction in mouse [16,17], *Drosophila* [35], and human neuronal [36,37] PKAN models.

In conclusion, the neuronal *Coasy* knock-out model provides an informative platform on which investigate the links between CoA and iron metabolisms, study the pathophysiology of CoA metabolism, and test potential therapeutic approaches.

## 4. Materials and Methods

### 4.1. Generation of Coasy^flox/flox^, +/Syn1-Cre (aka Syn-Coasy) Mice

The Coasy targeting vector, obtained from the European Mutant Mouse Consortium (EUCOMM) (ETPG00282_Y_2_E03), includes a lacZ/neo cassette with the β-galactosidase (lacZ) reporter and the neomycin (neo) resistance gene under the control of mouse En2 and human β-actin promoter, respectively (Figure S9). This cassette is flanked by two FRT sites (green) and is designed to be inserted on murine chromosome 11, upstream Coasy exon 2. A fragment containing mouse Coasy exons 2–9 is flanked by two loxP sites (red) (Figure 1A). The targeting vector was used to electroporate murine ES cells. ES cell DNA was extracted and digested with the restriction enzymes KpnI and EcoRV and then hybridized with 5′ and 3′ probes to check for recombination at the 5′ and 3′, respectively. The sizes of the expected fragments are shown for both the recombined and the not-recombined clones (Figure 1A). A supplementary neo-specific probe was used to confirm positive clones on KpnI-digested DNA. Southern blot analysis showed which clones were correctly recombined (Figure 1B). One homologous recombinant ES clone was microinjected into blastocysts from 129 J mice. The resulting chimeras were then bred with C57BL/6N mice to generate germ line-transmitted heterozygous mice (*Coasy^+/neo^*) (Figure 1A). To remove the neo cassette, *Coasy^+/neo^* mice were crossbred with transgenic mice expressing the transgene Flpe under the CMV promoter to generate *Coasy^+/flox^* mice (Figure 1A). Male *Coasy^+/flox^* mice were crossed with female transgenic mice expressing the Cre transgene under the rat Syn1 promoter (Jackson Laboratory, Bar Harbor, ME, USA; stock no. 003966), to obtain the *Coasy^+/flox^, +/Syn1-Cre* mice. Finally, female *Coasy^+/flox^,+/Syn1-Cre* mice were crossed with male homozygous *Coasy^flox/flox^* in order to obtain the conditional *Coasy^flox/flox^,+/Syn1-Cre* (Syn-Coasy) mice. PCR analysis was used for mice genotyping, using the primers listed in Table S1. A 596-bp product indicated the presence of the wild-type (*Coasy^+^*) allele, a 794-bp product indicated the presence of the floxed (*Coasy^flox^*) allele (Figure 1C), while a 350-bp product indicated the presence of Cre transgene (not shown). To visualize Cre expression, *+/Syn1-Cre* mice were crossed with a R26-stop-YFP (Jackson Laboratory, Bar Harbor, ME, USA; stock no. 006148).

### 4.2. Animal Studies

Animal studies were approved by the Italian Ministry of Health (Authorization No. 706/2017-PR, 14/09/2017), in accordance with the Italian Law D.L. 26/2014 and EU directive 2010/63/EU. The mice were kept on a C57Bl6/129Sv mixed background, and heterozygous littermates were used as controls. The animals were maintained in a temperature (21 ± 2 °C) and relative humidity (55 ± 10%) controlled animal-care facility with a 12 h light/dark cycle and free access to water and food. Euthanasia was carried out by cervical dislocation. Organs were quickly excised from euthanized animals and immediately flash frozen in liquid nitrogen (for molecular and biochemical analysis) or post-fixed in paraformaldehyde 4% for 24 h at 4 °C, cryoprotected in PBS containing 30% sucrose for 48 h at 4 °C, and then frozen in isopentane (for histological analysis and TOF-SIMS imaging).

Sensorimotor activity was assessed by surface righting and negative geotaxis tests as previously described [38].

### 4.3. Real-Time Quantitative PCR

Total DNA was extracted by the standard phenol-chloroform method. Total RNA was extracted with TRIzol reagent (Life Technology, Monza, Italy), retrotranscribed into cDNA, and qPCR was performed in duplicate using the GoTaq 2-step RT-qPCR system (Promega, Milan, Italy) according to the manufacturer’s protocols. All of the values were compared using the ΔΔCq method and the amounts of target DNA or cDNA (2^−ΔΔCq^) were calculated relative to the RNAseP or HPRT genes, respectively. All the primers used are listed in Table S1.

### 4.4. Immunoblotting

Pre-frozen mouse tissues were homogenized in 15 volumes of RIPA buffer (Tris-HCl 50 mM pH 7.5, NaCl 150 mM, EDTA 5 mM, NP40 1%, SDS 0.1%, and sodium deoxycholate 0.5%) in the presence of protease inhibitors. Homogenates were incubated on ice for 30 min, centrifuged at 10,000× *g* at 4 °C for 10 min, then the protein concentration was determined by Bio-Rad protein assay dye reagent (Bio-Rad, Segrate, Italy). In total, 20 to 50 µg of protein were run through SDS-PAGE and electroblotted onto a nitrocellulose membrane, which was then immunodecorated with the antibodies listed in Table S2. Band densitometry was carried out in ImageJ software (NIH, Bethesda, MD, USA).

### 4.5. CoAs and Pantothenic Acid Quantification by LC-MS/MS

For coenzyme A (CoA), acetylCoA (AcCoA), dephosphoCoA (dpCoA), and pantothenic acid measurements, tissues and extraction solvent were maintained on dry ice for the whole extraction process. Tissues were resuspended in 200 μL of methanol/water (80:20) containing 50 ng of hopantothenic acid (Hopan) as an internal standard. Samples were homogenized with tissue lyser for 1 min at max frequency and spun at 15,000× *g* for 10 min at 4 °C. Then, supernatants were passed through a regenerated cellulose filter, dried under N_2_ atmosphere, and resuspended in 100 μL of mobile phase (95% MeOH, 5% H_2_O + 5 mM NH_4_HCOO pH 7.5) for subsequent analysis. Quantification was performed through an API-4000 triple quadrupole mass spectrometer (AB Sciex, Milan, Italy) coupled with an HPLC system (Agilent, Milan, Italy) and CTC PAL HTS autosampler (CTC Analytics AG, Zwingen, Switzerland). The mobile phases were phase A: water + 5 mM NH_4_HCOO pH 7.5 and phase B: MeOH. A cyano-phase LUNA column (50 mm × 4.6 mm, 5 μm; Phenomenex, Bologna, Italy), in isocratic condition of 5% A and 95% B, with a flow rate of 800 μL/min was used. Resuspended samples were analyzed by a 5-min run in positive ion mode, and Multiquant software (version 3.0.2, AB Sciex, Milan, Italy) was used for data analysis and peak review of chromatograms. Quantitative analysis was achieved based on calibration curves for each analyte, and the total amount of metabolites was normalized on tissue weight.

### 4.6. TOF-SIMS Imaging

First, 12-μm-thick sagittal sections of the control and Syn-Coasy mice brain were cut at −20 °C with a MICROM HM505E cryostat microtome. The sections were immediately thaw mounted onto ITO-coated glass slides (Sigma-Aldrich, St Quentin Fallavier, France) and dried for 30 min in a desiccator under low vacuum before being placed in a plastic bag filled with N_2_ and stored at −80 °C until analysis. Tissue sections were washed with methanol to avoid the interference of lipids in the detection of metal ions. The sections were then incubated at 100 °C in an oven for approximately 6h to realize ferritin denaturation. In general, ferritin shows high thermal stability and most often the effective unfolding takes place at >80 °C [39,40]. The release of the caged Fe in ferritin after heat treatment has been demonstrated previously by SIMS analysis [41].

The imaging experiments here were performed on a TOF-SIMS IV (IONTOF GmbH, Münster, Germany) instrument. The liquid metal ion gun (LMIG) was operated in high current bunch (HCBU) mode and 25 keV Bi_3_^+^ cluster ions were selected as the primary ion beam. The beam current was about 0.45 pA measured at 10 kHz. The secondary ions were extracted and accelerated to 2 keV at the entrance of the TOF analyzer, and then post-accelerated to 10 keV before reaching the detector. A low-energy pulsed electron flood gun (21 eV) was employed to compensate the charge accumulation on the insulating tissue surface. The whole sagittal tissue section was imaged by moving the sample stage via patches of 500 µm × 500 µm with a pixel size of 31.25 µm and an ion dose of 5.6 × 10^9^ ions/cm^2^. For regional mapping of the cortex and medulla with a high spatial resolution of 2 µm, the ion images were generated from areas of 500 µm × 500 µm divided by 256 × 256 pixels, with a total ion dose of ~5 × 10^11^ ions/cm^2^. Data processing was performed using SurfaceLab 7 software (IONTOF GmbH, Münster, Germany). Mass spectra were acquired in positive ion mode and internally calibrated using small fragments commonly observed in SIMS spectra, such as CH^+^, CH_2_^+^, CH_3_^+^, C_2_H_3_^+^, and C_2_H_5_^+^.

### 4.7. Histology, Immunohistochemistry, and Immunofluorescence

Brain sections of 30 μm were cut with a cryostat (Leica Biosystems, Buccinasco, Italy) and stored in PBS plus 0.1% sodium azide at 4 °C. Sections were mounted on gelatin-coated glass slides, air-dried, and stained with haematoxylin-eosin, thionine, and Perls’ stains, following standard procedures.

For immunostaining, brain sections were incubated in H_2_O_2_ 0.1% for 10 min to deactivate endogenous peroxidases, washed in PBS, and incubated in blocking solution (PBS with 10% FBS and 0.3% Triton X-100) for 1 h at room temperature. Then, slices were incubated with different primary antibodies (listed in Table S2) diluted in blocking solution. For immunohistochemistry (IHC), goat anti-rabbit or anti-mouse biotinylated secondary antibodies (1:1000, Jackson Immunoresearch, Ely, UK) were applied for 1 h at room temperature. Signal detection was performed by using Vectastain Elite ABC kit (Vector, Burlingame, CA, USA) and DAB solution (Vector, Burlingame, CA, USA), according to the manufacturer’s instructions. Sections were mounted on gelatin-coated glass slides with cromalin solution, dehydrated in graded ethanol, cleared in Bioclear (Bio-Optica, Milan, Italy), and covered with DPX (Sigma-Aldrich, Milan, Italy). Labeled structures were examined under bright-field illumination on a Zeiss Axioplan2 microscope (Zeiss, Milan, Italy). Digital images were acquired with a Zeiss camera using AxioVision software (Zeiss, Milan, Italy). Percentage of positive area was calculated using ImageJ software (NIH, Bethesda, MD, USA). For immunofluorescence (IF), sections were stained with Alexa Fluor 488-conjugated goat anti-rabbit IgG (Thermo Fisher, Monza, Italy), Alexa Fluor 568-conjugated goat anti-mouse IgG (Thermo Fisher, Monza, Italy), Neurotrace fluorescent Nissl stain (ThermoFisher, Monza, Italy), and TO-PRO-3 nucleic acid stain (ThermoFisher, Monza, Italy). Sections were mounted on gelatin-coated glass slides using Fluorsave (Calbiochem, San Diego, CA, USA) and acquired on a TCS-SP8 laser confocal microscope using LAS AF software (Leica Biosystems, Buccinasco, Italy).

### 4.8. Determination of Heme Content

Heme content was measured by a spectrophotometric method as previously described [42]. Liquid nitrogen-frozen tissues were dissolved in 50 volumes (w/v) of 98% formic acid and incubated for 15 min at 37 °C. After centrifugation (8000× *g* 10 min), heme content was evaluated by analyzing clear supernatant at 400 nm. The standard curve was obtained using Hemin (Sigma-Aldrich, Milan, Italy). The data were normalized to the protein content measured by the Bio-Rad Protein Assay (Bio-Rad, Segrate, Italy).

### 4.9. Evaluation of Mitochondrial Bioenergetics

For individual respiratory complexes analysis, liquid nitrogen-frozen tissues were homogenized in 15 volumes (w/v) of potassium-phosphate buffer 10 mM pH 7.4, and biochemical assays of the mitochondrial respiratory complexes and of citrate synthase (CS) were carried out as described [43].

For global mitochondrial respiration analysis, we measured oxygen consumption on isolated mitochondria derived from the mouse brain using an XF96 Extracellular Flux Analyzer (Agilent, Milan, Italy) as described [16,44]. Briefly, to rapidly isolate mitochondria, brains were obtained from sacrificed mice and homogenized in ice-cold mitochondrial isolation buffer (mannitol 210 mM, sucrose 70 mM, HEPES 5 mM, EGTA 1 mM, 0.5% fatty acid-free BSA, pH 7.2) using a glass-dounce homogenizer. The homogenate was centrifuged for 10 min at 800× *g* at 4 °C and the supernatant was collected and centrifuged for 10 min at 8000× *g* at 4 °C. The pellet was then washed twice and finally resuspended in mitochondrial assay solution (mannitol 220 mM, sucrose 70 mM, KH_2_PO_4_ 10 mM, MgCl_2_ 5 mM, HEPES 2 mM, EGTA 1 mM, 0.2% fatty acid-free BSA, pH 7.2) supplemented with complex I respiratory substrates (pyruvate 10 mM, malate 5 mM). Mitochondria were seeded in an XF 96-well cell culture microplate (Agilent, Milan, Italy) at a protein concentration of 10 µg/well, the plate was centrifuged for 20 min at 2000× *g* at 4 °C, and then incubated for 15 min at 37 °C without CO_2_. OCR was measured under basal conditions (state II), and after sequentially adding to each well 20 µL of ADP (state III), 22 µL of oligomycin (state IVo), 24 µL of carbonyl cyanide-4-(trifluoromethoxy)phenylhydrazone (FCCP) (state IIIu), and 26 µL of rotenone, to reach a working concentration of 4 mM, 2.5 μg/mL, 3.9 μM, and 1.9 μM, respectively.

### 4.10. Electron Microscopy

The samples were fixed for 24 h at 4 °C with 4% paraformaldehyde and 2.5% glutaraldehyde in 125 mM cacodylate buffer. Then, they were post-fixed (1 h) with 2% OsO4 in 125 mM cacodylate buffer, and washed and embedded in Epon. Conventional thin sections were collected on uncoated grids, stained with uranil and lead citrate. Grids were examined with a Talos L 120C electron microscope (Thermo Scientific, Monza, Italy) at 120 kV.

### 4.11. Determination of Aconitase Activity

Aconitase activity was in-gel assayed as described [45]. Tissues were homogenized in 15 volumes (w/v) of Lysis buffer (Tris-HCl 20 mM pH 7.4, Triton X-100 1%, citrate 2 mM, MnCl_2_ 0.6 mM, KCl 40 mM) in the presence of protease inhibitors. After centrifugation, 30 μg of cleared supernatant were added to the loading buffer (Tris-HCl 25 mM, 10% glycerol, bromophenol blue) and run in an 8% acrylamide native gel at 4 °C. Aconitase activity was determined in the dark at 37 °C by incubating the gel in Tris-HCl 100 mM pH 8.0, NADP 1 mM, cis-aconitic acid 2.5 mM, MgCl_2_ 5 mM, MTT 1.2 mM, phenazine methosulfate 0.3 mM, and isocitrate dehydrogenase 5 U/mL. The signal quantification was carried out using ImageJ software (NIH, Bethesda, MD, USA).

### 4.12. Statistical Analysis

All the experiments were performed at least in triplicate. Data were analyzed with GraphPad Prism 8, using two-tailed unpaired Student’s *t*-test and one- or two-way ANOVA followed by Bonferroni post-hoc test. The data are reported as the mean ± SD. The *p*-value < 0.05 was considered statistically significant.

## Supplementary Materials

The following are available online at https://www.mdpi.com/1422-0067/21/24/9707/s1.

## Abbreviations

CoA	Coenzyme A
COASY	Coenzyme A synthase
CoPAN	COASY protein-associated neurodegeneration
dpCoA	Dephospho-coenzyme A
GP	Globus pallidus
NBIA	Neurodegeneration with brain iron accumulation
PANK	Pantothenate kinase
PKAN	Pantothenate kinase-associated neurodegeneration
TOF-SIMS	Time of flight-secondary ion mass spectrometry
