# Supplementary material for: Neuronal Ablation of CoA Synthase Causes Motor Deficits, Iron Dyshomeostasis, and Mitochondrial Dysfunctions in a CoPAN Mouse Model
Source: Int J Mol Sci. 2020 Dec 19;21(24):9707. doi: 10.3390/ijms21249707 (PMC7766928; doi:10.3390/ijms21249707)
Supplement: Supplementary file 1 [file ijms-21-09707-s001.zip › ijms-1034704 supplementary.pdf]

Neuronal Ablation of CoA Synthase Causes Motor Deficits, Iron Dyshomeostasis and Mitochondrial Dysfunctions in a CoPAN Mouse Model

Ivano Di Meo, Chiara Cavestro, Silvia Pedretti, Tingting Fu, Simona Ligorio, Antonello Manocchio, Lucrezia Lavermicocca, Paolo Santambrogio, Maddalena Ripamonti, Sonia Levi, Sophie Ayciriex, Nico Mitro and Valeria Tiranti

Supplementary figures and tables

Table of contents:

- **Supplementary Figure S1:** Syn1-Cre recombinase activity in brain.
- **Supplementary Figure S2:** Coasy protein in extra-cerebral organs.
- **Supplementary Figure S3:** Immunohistochemical analysis of astrocytes and microglia.
- **Supplementary Figure S4:** Ferritin quantification
- **Supplementary Figure S5:** Accumulation of Fe, Mg and Ca in Syn-Coasy mouse cortex.
- **Supplementary Figure S6:** Analysis of proteins involved in autophagy-lysosomes.
- **Supplementary Figure S7:** Analysis of lipoylated proteins.
- **Supplementary Figure S8:** Oxidative stress analysis.
- **Supplementary Figure S9:** Coasy targeting vector.
- **Supplementary Table S1:** List of PCR and qPCR primers.
- **Supplementary Table S2:** List of antibodies

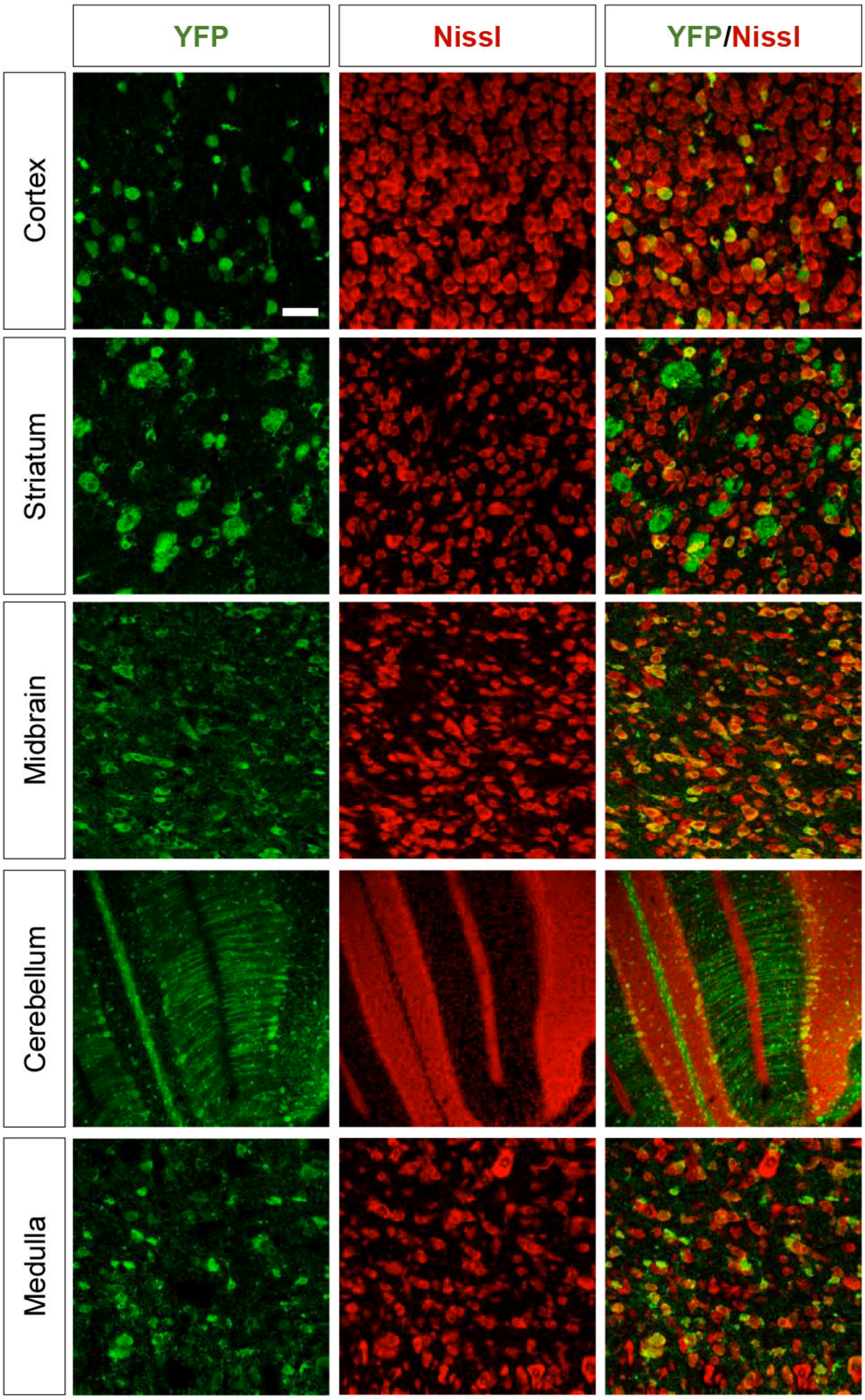

Supplementary Figure S1. Syn1-Cre recombinase activity in brain. Syn-Cre transgenic mouse was crossed with R26-stop-YFP reporter mice. Confocal immunofluorescence performed with a YFP antibody (green) and neuronal Nissl staining (red) showed Cre recombinase specifically activated in neurons through all the brain. Scale bar: 25 μm.

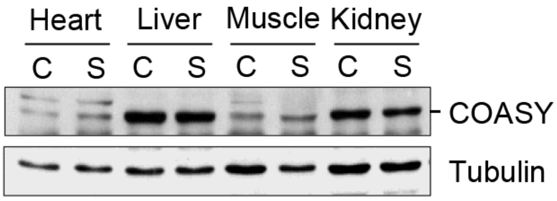

Supplementary Figure S2: Coasy protein in extra-cerebral organs. Western blot analysis of COASY protein in Ctrl (C) and Syn-Coasy (S) extra-CNS tissues.

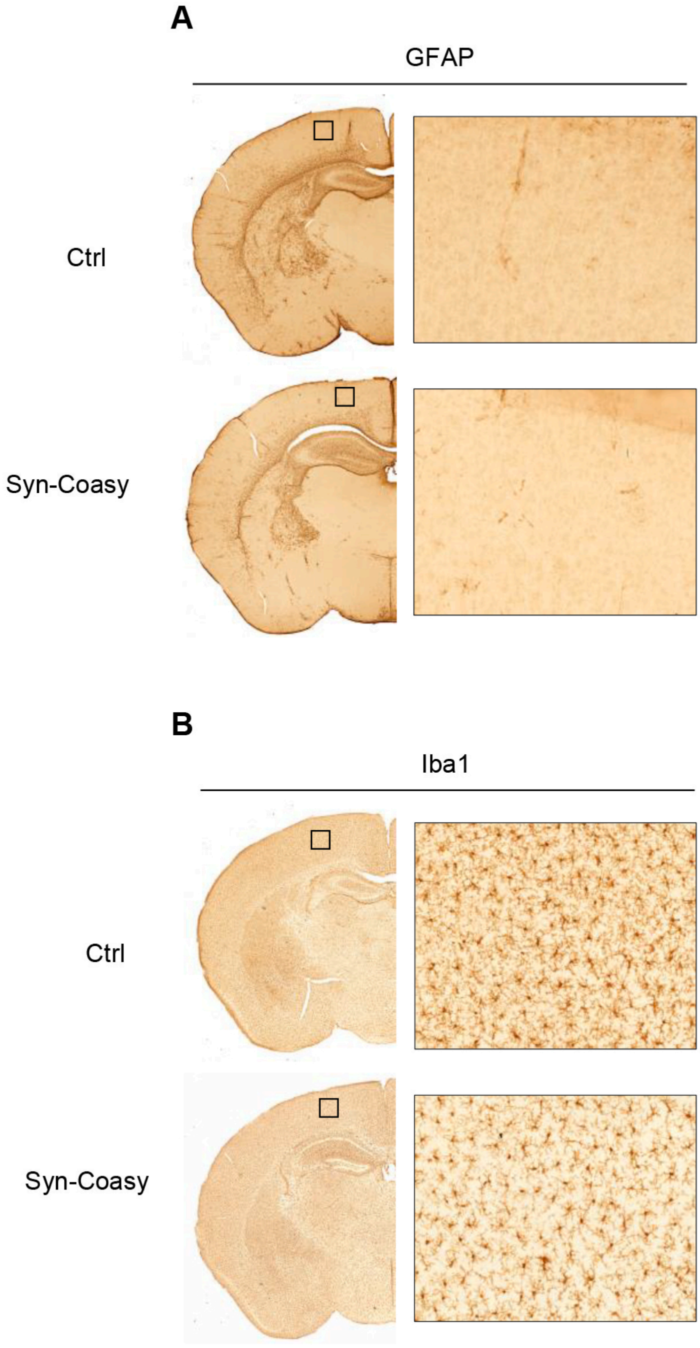

Supplementary Figure S3. Immunohistochemical analysis of astrocytes and microglia. Coronal sections immunolabeled with antibodies specific for (**A**) GFAP and (**B**) Iba1 showing absence of astrocytosis and neuroinflammation in Syn-Coasy brain (bottom panels) compared to control littermates (top panel).

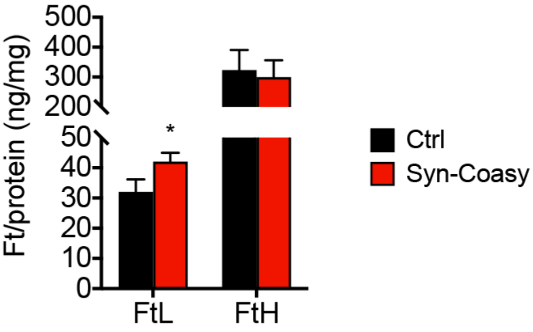

Supplementary Figure S4: Ferritin quantification. The soluble forebrain homogenate fractions of Syn-Coasy and Ctrl mice were analyzed for L- and H-ferritins (FtL and FtH, respectively) content by specific ELISA. Mean ± SD is showed. * $p < 0.05$ (one-way ANOVA).

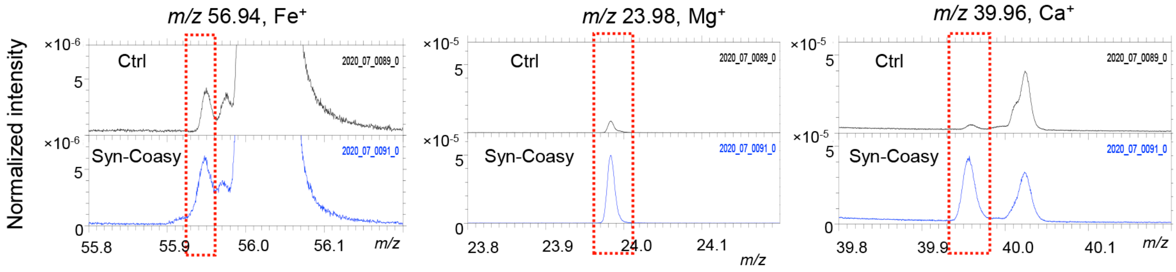

Supplementary Figure S5: Accumulation of Fe, Mg and Ca in Syn-Coasy mouse cortex. Mass spectra obtained from the cortex regions of control (Ctrl) and Syn-Coasy mice brain, showing the elevation of Fe, Mg and Ca in Syn-Coasy.

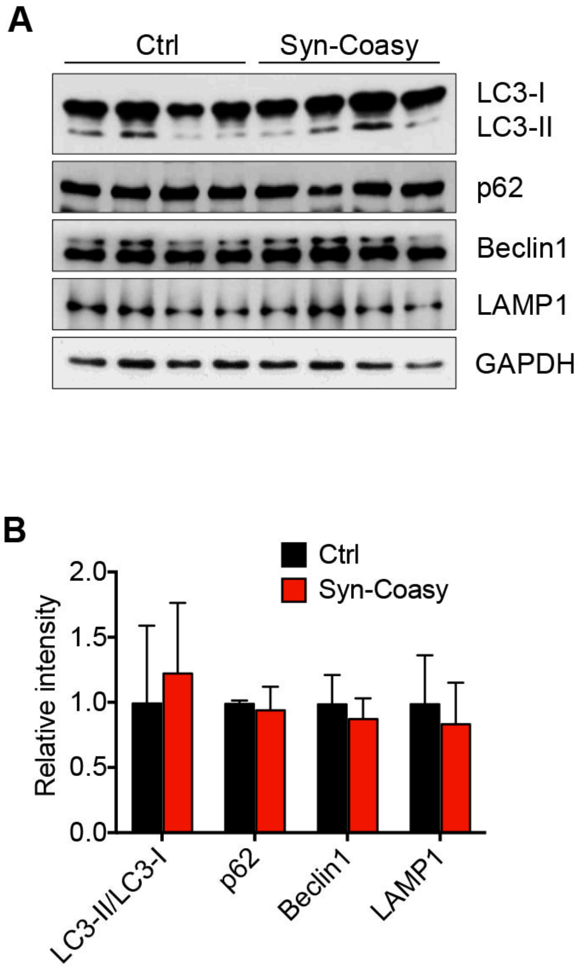

Supplementary Figure S6. Analysis of proteins involved in autophagy-lysosomes. (**A**) Western blot analysis and (**B**) densitometric quantification of proteins associated to autophagosomes and lysosomes in forebrain homogenates from Ctrl (n = 8) and Syn-Coasy (n = 8) mice. GAPDH was used as loading control. No statistically significant differences were observed (one-way ANOVA).

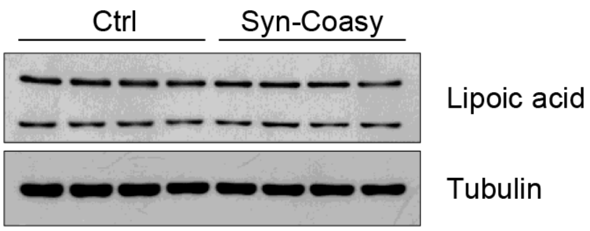

Supplementary Figure S7. Analysis of lipoylated proteins. Western blot analysis using an anti-lipoic acid antibody showing no differences in the level of lipoylated proteins in Syn-Coasy forebrain (n = 8) compared to control littermates (n = 8).

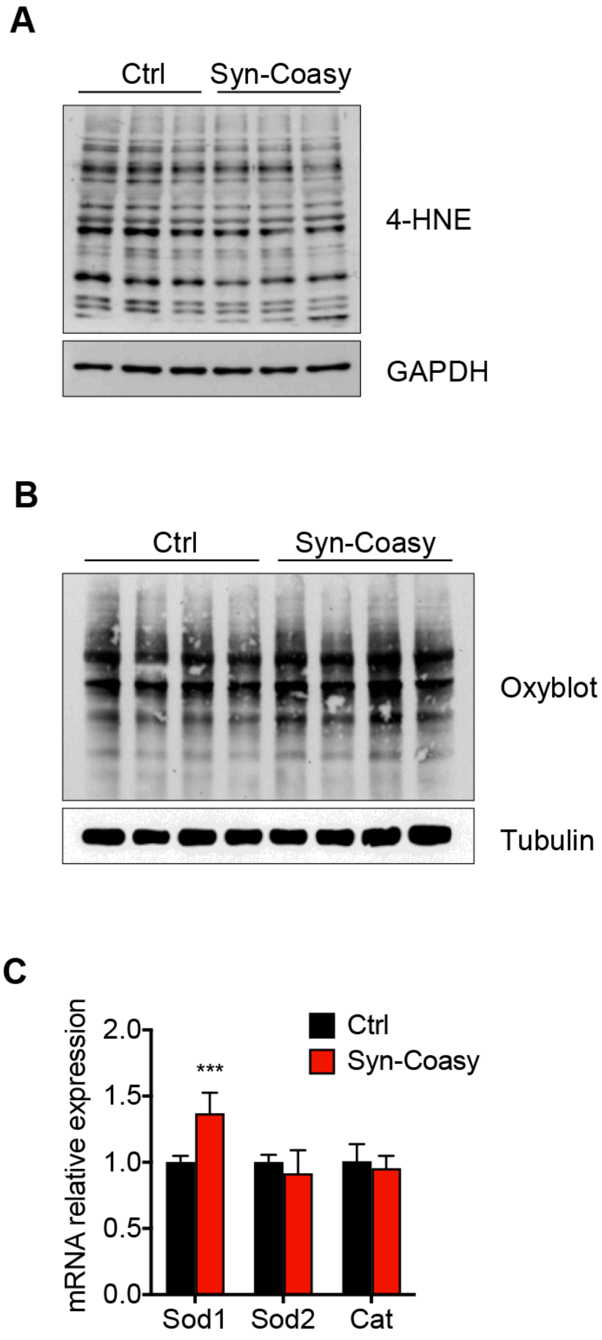

Supplementary Figure S8. Oxidative stress analysis. (**A**) 4-HNE immunoblot and (**B**) oxyblot analysis of forebrain lysates showed similar levels of both lipids and protein peroxidation in Syn-Coasy mice compared to control littermates. (**C**) Relative Sod1, Sod2 and Cat mRNA expression in Ctrl (n = 8) and Syn-Coasy (n = 8) mice forebrain. Mean ± SD is showed. *** $p < 0.001$ (one-way ANOVA).

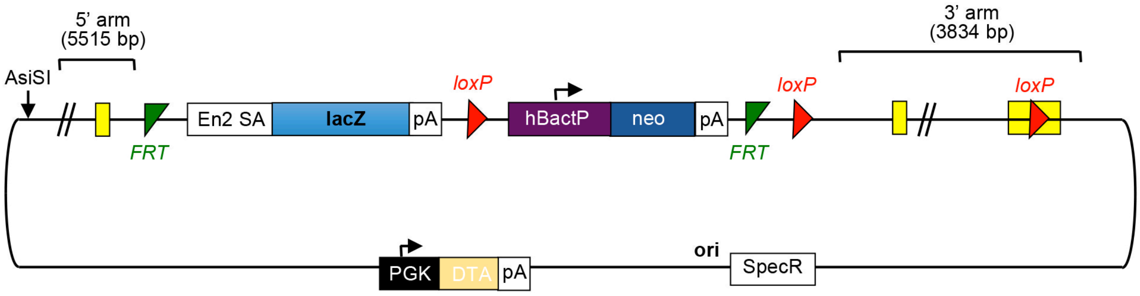

Supplementary Figure S9. Coasy targeting vector. Schematic representation of ETPG00282_Y_2_E03 vector (EUCOMM) used for homology recombination. The vector is composed by a cassette with the β-galactosidase (lacZ) reporter and the neomycin (neo) resistance gene under the control of mouse En2 and human β-actin promoter respectively, flanked by the Flpe recombinase-specific FRT sites (green). The cassette is designed to be inserted upstream Coasy exon 2. Cre-specific loxP sites are in red.

Supplementary Table S1. PCR and qPCR primers

Use	Gene	Forward primer (5′- 3′)	Reverse primer (5′- 3′)
Genotyping	Coasy	AGTTCTTGGGGGTTCTCCAC	CTCGCCTCCACAGACAAGTT
Genotyping	Cre	CGCAGAACCTGAAGATGTTC	GTTCGAACGCTAGAGCCTG
DNA-qPCR	Coasy	ATCTAATGGTGAGGAGAA	GAGGGTAATGAACTGATG
RT-qPCR	Coasy	TGCTTCAGCCTCCAAATGA	CTTTTCCCAGAGCCACTGAT
RT-qPCR	Pank1α	GTTCGCCCAGCATGATTCTC	CTTAACCAGGGTTCCACCGAT
RT-qPCR	Pank1β	CTGAGCCTAACTCCATTCAACT	TCCACCGATATCCATACCAAAC
RT-qPCR	Pank2	TTGGGCATACGTGGAGCTTT	TCTCACATACATTTCAACAGGA
RT-qPCR	Pank3	AACCTCCACCTGCACAAACT	TGGGTAAGGATCATCCAGGT
RT-qPCR	Ppcs	CTCTCAGTCCATTAGGCTCTTC	GGATCTTGTGTTCAGGCATTTC
RT-qPCR	Ppcdc	TAACAACAGAGAGAGCCAAACA	GCTTCCACATCTCCCATTCA
RT-qPCR	Ftl	TGAACCGCCTGGTCAACT	CGGAAGAAGTGGCCTACG
RT-qPCR	Fth1	TGGAGTTGTATGCCTCCTACG	TGGAGAAAGTATTTGGCAAAGTT
RT-qPCR	Tfr1	TCCTTTCCTTGCATATTCTGG	CCAAATAAGGATAGTCTGCATCC
RT-qPCR	Dmt1	CTCCACCATGACTGGAACCT	TTCAGGAATCCCTCCATGAC
RT-qPCR	Fpn	ACCCATCCCCATAGTCTCTGT	ACCGTCAAATCAAAGGACCA
RT-qPCR	Sod1	CAAGCGGTGAACCAGTTGTG	TGAGGTCCTGCACTGGTAC
RT-qPCR	Sod2	GCCTGCACTGAAGTTCAATG	ATCTGTAAGCGACCTTGCTC
RT-qPCR	Cat	TGGCACACTTTGACAGAGAGC	CCTTTGCCTTGGAGTATCTGG

Supplementary Table S2. Antibodies

Antibody	Host animal	Dilution (WB)	Dilution (IHC/IF)	Source (Catalog #)
4-HNE	Rabbit	1:2000		Abcam (ab46545)
acH3	Rabbit	1:10000		Millipore (06-599)
acLys	Rabbit	1:1000		Cell Signaling (9441)
acTUB	Mouse	1:20000		Sigma-Aldrich (T7451)
Beclin	Rabbit	1:1000		Cell Signaling (3738)
CB	Mouse		1:5000	Swant (300)
COASY	Rabbit	1:2000		ThermoFisher (PA5-28696)
CR	Mouse		1:2000	Swant (6B3)
DMT1	Rabbit	1:100		Santa Cruz (sc-166884)
Fpn	Rabbit	1:1000		Homemade[1]
FtH	Rabbit	1:1000	1:700	Homemade[2]
FtL	Rabbit	1:1000	1:700	Homemade[2]
GAPDH	Mouse	1:1000		Millipore (MAB374)
GFAP	Rabbit		1:1000	Millipore (AB5804)
GFP	Rabbit		1:1000	Abcam (ab290)
Iba1	Rabbit		1:1000	Wako (019-19741)
LAMP1	Rabbit	1:1000		Sigma-Aldrich (L1418)
LC3	Rabbit	1:1000		Cell Signaling (2775)
Lipoic acid	Rabbit	1:1000		Abcam (ab58724)
NeuN	Mouse		1:1000	Millipore (MAB377)
P62	Rabbit	1:1000		Sigma-Aldrich (P0067)
PV	Mouse		1:5000	Swant (PV235)
TfR1	Mouse	1:1000		ThermoFisher (13-6800)
Tubulin	Mouse	1:2000		Sigma-Aldrich (T0198)
